# The Prevalence of Depression, Anxiety and Stress and Their Associated Factors in College Students

**DOI:** 10.3390/ijerph17197001

**Published:** 2020-09-24

**Authors:** Enrique Ramón-Arbués, Vicente Gea-Caballero, José Manuel Granada-López, Raúl Juárez-Vela, Begoña Pellicer-García, Isabel Antón-Solanas

**Affiliations:** 1 Faculty of Health Sciences, Campus Universitario Villanueva de Gállego, Universidad San Jorge, 50830 Villanueva de Gállego, Zaragoza, Spain; eramon@usj.es; 2 Nursing School La Fe, Adscript Center of University of Valencia, 46026 Valencia, Spain; 3 Research Group GREIACC, Health Research Institute La Fe, 46026 Valencia, Spain; 4 Faculty of Health Sciences, Zaragoza University, 50009 Zaragoza, Spain; 5 Faculty of Health Sciences, La Rioja University, 26006 Logroño, Spain; raul.juarez@unirioja.es; 6 Servicio Aragonés de Salud, Sector Alcañiz Atención Primaria, Centro de Salud Andorra Calle Huesca, 44500 Andorra, Spain; beg2008@hotmail.es; 7 Department of Physiatry and Nursing, Faculty of Health Sciences, University of Zaragoza, 50009 Zaragoza, Spain; ianton@unizar.es

**Keywords:** depression, anxiety, stress, psychological, student health, Spain, cross-sectional studies

## Abstract

Aim: To estimate the prevalence of symptoms of depression, anxiety, stress and associated factors in a population of college students. Method: Cross-sectional study of psychological distress measured through the Depression, Anxiety and Stress Scale (DASS-21) in a sample of 1074 college students. Results: We found a moderate prevalence of depression (18.4%), anxiety (23.6%) and stress (34.5%) symptoms in our study population. Being <21, having problematic Internet use behavior, smoking, presenting insomnia and having a low self-esteem were independently associated with symptoms of depression, anxiety and stress. Being a woman, living with their family, having a stable partner, consuming alcohol frequently and having poor nutritional habits were significantly associated with symptoms of stress; lacking a stable partner was significantly associated with depressive symptoms; and frequent consumption of alcohol was significantly associated with symptoms of anxiety. Conclusion: We found a moderate prevalence of depression, anxiety and stress symptoms in our population. Interventions aimed at promoting mental health among college students should be implemented.

## 1. Introduction

In its plan for the prevention, treatment and overcoming of mental health disorders, the World Health Organization described mental health as fundamental to human health [1]. Yet, mental health problems are the first cause of disability and a major public health issue worldwide due to disease progression, difficulties in therapeutic management and increasing prevalence [2,3]. Specifically, depression, anxiety and stress are considered important indicators for mental health which, if untreated, can have a negative effect on individuals [4,5]. According to the American Psychological Association, anxiety and depression are both emotional responses leading to a very similar set of symptoms, including difficulty sleeping, fatigue, muscle tension and irritability. Whereas stress is usually caused by an external factor and can be short-term, anxiety is persistent, even in the absence of a stressor [6]. Depression is characterized by a set of symptoms including a lack of interest in daily activities, significant weight loss or gain, sleep pattern alterations, lack of energy, loss of concentration, feelings of worthlessness or guilt and even recurrent thoughts of death or suicide [7].

Most mental health problems appear by early adulthood, yet young adults rarely get any support for their mental health [8]. Furthermore, mental health issues in this population are associated with higher incidence of physical and emotional problems in the mid to long term [9], labor market marginalization [10], worse quality of sleep [11] and dysfunctional relationships [12], among others. College students are at risk of experiencing stress, anxiety and depression, which cause psychological distress and may impact on their academic performance [13]. Worldwide, it is estimated that 12–50% of college students present at least one diagnostic criterion for one or more mental disorders [14]. Causes of stress during college life include academic pressure stemming from factors such as exams and workload, lack of leisure time, competition, concerns about not meeting parents’ expectations, establishing new personal relationships and moving to a strange location [15]; biological factors such as age and gender, specifically being female [16]; and financial burden [17].

Globally, studies conducted on different samples of undergraduate students have identified a moderate to high prevalence of depression, anxiety and stress in this population [18,19,20,21,22,23]. Early diagnosis and management of psychological distress lead to better management and patient outcomes [24]. Thus, it is necessary to identify those students who are at a higher risk of developing mental health problems during college life.

In Spain, mental health problems are highly prevalent in the general population [25], as well as in specific groups [26,27]. However, little is known about the mental health of college students. Previous studies have reported a high prevalence of anxiety and depression in this population [28,29], but sample size was small and they did not measure stress. Furthermore, since psychological health status was not the main research variable, predictive factors were not reported. Based on the above, we aim to determine the prevalence of anxiety, depression and stress, and their associated factors in a sample of Spanish college students.

## 2. Materials and Methods

### 2.1. Design

A cross-sectional study of the prevalence of symptoms of depression, anxiety, stress and associated factors was carried out in a population of college students registered in San Jorge University (SJU) in Zaragoza (Spain).

### 2.2. Sample

Our participants were undergraduate students registered in one of the bachelor’s degrees offered by SJU’s Faculty of Health Sciences, Faculty of Communication and School of Architecture and Technology during the academic year 2018–2019. Participant recruitment took place from September 2018 to May 2019. The students were informed about the aims of the study and the methods of data collection by a researcher in the classroom; a copy of the participant information leaflet and consent form was given to the students at this time. The students were assured that privacy and confidentiality would be maintained, and that they had a right to refuse to participate in the study or to withdraw consent to participate at any time without reprisal. Of a total population of 1341, 1074 students gave their consent to participate in this study and completed the questionnaire.

### 2.3. Data Collection

The questionnaire was divided into two sections, namely sociodemographic characteristics (including anthropometry and habits) and psychological health. Sociodemographic data included a list of variables generally associated with psychological distress in younger populations [20,21], namely age, gender, bachelor’s degree, place of residence, personal relationship, height, weight, financial status, tobacco and alcohol consumption, physical activity, diet and Internet use. As in a previous study by Mahroon et al. [30], the variable age was dichotomized into <21 and ≥21. This allowed us to estimate differences in the prevalence of anxiety, stress and depression in relation to the students’ age group and year of study. The variable body mass index (BMI) was calculated from weight and height self-report (BMI = kg/m^2^). BMI was trichotomized to: (1) low BMI (≤18.5 kg/m^2^), (2) normal BMI (18.5–24.9 kg/m^2^) and (3) high BMI/obese (≥25 kg/m^2^).

Physical activity was measured using the short form of the International Physical Activity Questionnaire (IPAQ-SF). This tool measures the intensity, frequency and duration of physical activity over the last seven-day period. Metabolic equivalents (METs), defined as “the ratio of a person’s working metabolic rate relative to their resting metabolic rate” [31], are then calculated for each of the physical activities undertaken including walking, moderate and vigorous physical activity. The results are then added up to obtain a measure of the total physical activity undertaken in the previous seven days and the participants are subsequently classified into one of three categories of physical activity (low, medium and high) [32].

Internet use was measured using Young’s Internet Addiction Test (IAT). This tool comprises 20 items answered on a 5-point Likert scale ranging from 1 to 5, indicating the extent to which they endorse each particular behavior. The IAT total score ranges from 0 to 100, where higher scores represent higher levels of severity of Internet compulsivity and addiction. Specifically, scores <50 suggest a controlled use of the Internet, scores between 50 and 79 suggest excessive Internet use and scores ≥80 imply that the participant is experiencing severe Internet addition, which impacts on his or her personal as well as social life [32]. In this study, Internet use was analyzed as a dichotomous variable where IAT <50 indicated no problematic Internet use (PIU) and IAT ≥50 indicated PIU [33].

We assessed the quality of the participants’ diet through the Healthy Eating Index (HEI) in its Spanish version [34]. The HEI uses a scoring system ranging from 1 to 10 to evaluate a set of foods. Total score ranges from 0 to 100, with higher scores suggesting healthier dietary habits. HEI scores >80 indicate a good or healthy diet, scores ranging from 50 to 80 suggest a diet that needs improvement and scores <50 imply a poor or unhealthy diet.

The presence and severity of insomnia was assessed through the Insomnia Severity Index (ISI). The ISI is a 7-item self-report questionnaire assessing the nature, severity and impact of insomnia experienced in the last month. A 5-point Likert scale is used to rate each item (0 = no problem; 4 = very severe problem) yielding a total score ranging from 0 to 28. The ISI total score is interpreted as follows: absence of insomnia (0–7); sub-threshold insomnia (8–14); moderate insomnia (15–21); and severe insomnia (22–28) [35].

The participants’ self-esteem was assessed using the Rosenberg Self-Esteem Scale (RSES). This tool consists of 10 items, five of which are expressed in positive statements and the other five in negative statements. Negative items were reverse-scored prior to analysis. The RSES uses a 4-point response scale (1 = strongly disagree; 2 = disagree; 3 = agree; 4 = strongly agree) with total scores ranging from 10 to 40. Respondents are classified into three levels of self-esteem: high self-esteem (≥30 points), medium self-esteem (26–29 points) and low self-esteem (≤25 points) [36].

Finally, symptoms of anxiety, stress and depression were measured through the Depression Anxiety Stress Scales (DASS-21). The DASS-21 consists of 21 items, 7 items per subscale: DASS-D (depression), DASS-A (anxiety) and DASS-S (stress). Respondents must rate the extent to which each statement applies during the past week on a 4-point Likert scale ranging from 0 (did not apply to me at all) to 3 (applied to me very much). Because the DASS-21 is a short-form version of the DASS (42 items), the final score for each sub-scale is multiplied by two and evaluated according to its severity rating index. Depression, anxiety and stress scores are calculated by adding up the scores of the items in each separate subscale. The results are interpreted as follows: DASS-A (>19 = extremely severe depression; 19–15 = severe anxiety; 14–10 = moderate anxiety; 9–8 = mild anxiety; 7–0 = no anxiety/normal), DASS-D (>27 = extremely severe depression; 27–21 = severe depression; 20–14 = moderate depression; 13–10 = mild depression; 9–0 = no depression/normal), DASS-S (>33 = extremely severe stress; 33–26 = severe stress; 25–19 = moderate stress; 18–15 = mild stress; 14–0 = no stress/normal). This tool has been previously validated in a population of Spanish college students showing high levels of consistency for the three subscales [37].

### 2.4. Data Analysis

The sample characteristics were analyzed using frequency and percentage for qualitative variables and mean and standard deviation for quantitative ones. We used a Kolmogorov–Smirnov test to test for normality of distribution in our data. Bivariant analyses were carried out using Chi-Square, Mann–Whitney or t-test, as applicable. In addition, we carried out a binary logistic regression (backward stepwise method with a probability value for the entry of *p* = 0.05 and removal of *p* = 0.10) analysis in order to determine the predictive factors of psychological health in our sample (presence of anxiety, stress, depression). We performed a collinearity analysis in order to detect variables which showed a condition index ≥30. Subsequently, two analyses were carried out with and without outliers, with minimal differences between them. In this manuscript, we present the results from the analysis including outliers. Data codification, processing and analysis were completed using the statistical software Statistical Package for the Social Science (SPSS version 21 for Windows, IBM Corp., Chicago, IL, USA) accepting a level of significance of *p* < 0.05.

### 2.5. Ethical Considerations

This study was reviewed and approved by the Clinical Research Ethics Committee of Aragón (IRB Ref: CP-CI.PI09/93) prior to the start of this investigation. We confirm that each and every one of the national and international standards for ethical research with human subjects were respected and adhered to.

## 3. Results

A total of 1074 undergraduate students (71% women and 29% men) took part in this investigation. Age ranged from 18 to 42, with an average of 21.73 ± 5.12 years. The majority of our students were enrolled in a healthcare program (57.3%), had a normal BMI (69.8%), perceived their financial status to be medium (74.7%), did not have a stable partner (53.2%) and lived with their family (66.3%). In addition, 24.9% consumed tobacco habitually, 28% had a low level of physical activity, 23% experienced PIU, 42.9% had some degree of insomnia and 82.6% needed diet improvement (see Table 1).

Of our participants, 23.6% and 34.5% had symptoms of anxiety and stress above the normal range, respectively. In both cases, women’s levels of anxiety and stress were higher than men’s (*p* < 0.05). The symptoms of depression, on the other hand, were evenly distributed between our male and female participants (19.3% men and 18.1% women) (see Table 2).

**Figure 1:**
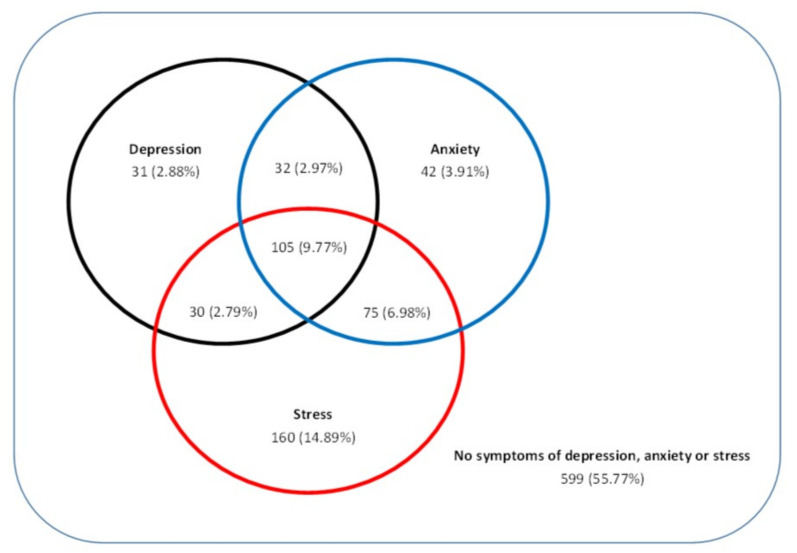
Coexistence of symptoms of depression, anxiety and stress according to the results from DASS-21 (*n* = 1074).

Of our participants, 22.5% presented symptoms of two mental disorders according to the DASS-21 questionnaire, and up to 9.7% of our sample experienced symptoms of anxiety, depression and stress simultaneously (see Figure 1).

We performed a collinearity analysis in order to detect variables which showed a condition index ≥30. None of them reached this value. Subsequently, two analyses were carried out with and without outliers, with minimal differences between them. In this manuscript, we present the results from the analysis including outliers. The final models of binary logistic regression evidenced that being <21, experiencing PIU, smoking, having insomnia and reporting a low level of self-esteem were associated with depression, anxiety and stress (*p* < 0.05). In addition, being a woman, living with their family, having a stable partner, consuming alcohol regularly and following an inadequate or unhealthy diet were significantly associated with stress; not having a stable partner was associated with depression; and frequent alcohol consumption was associated with anxiety (see Table 3).

## 4. Discussion

This, to our knowledge, is the first report of the prevalence of symptoms of anxiety, depression and stress, and their associated factors, in a sample of Spanish college students. Although the DASS-21 questionnaire cannot be considered as a tool for the diagnosis of psychological pathology, it is useful to identify the prevalence of symptoms of anxiety, depression and stress. We identified a significant prevalence of symptoms of stress (34.5%), anxiety (23.6%) and depression (18.4%) in our population. Previous studies carried out in Spain involving smaller samples have reported an even greater prevalence of psychological distress in our population [28,29]. Specifically, Balanza et al. [28] reported a prevalence of anxiety and depression of 41.7% and 55.6% respectively using the Goldberg Anxiety and Depression Scale. Fernández et al. [29] identified an even higher percentage of students with anxiety symptoms (44.7%) and a lower prevalence of depressive symptoms (23.5%) using the Hospital Anxiety and Depression Scale (HADS). Unfortunately, the use of different screening tools does limit the comparability of the findings. 

Worldwide, there is variation in the reported prevalence of psychological distress among college students. A systematic review of 24 studies estimated an average prevalence of depression of 30.5%, with results ranging between 10.4 and 80.5% [38]. The same level of variation was observed in previous studies which used the DASS questionnaire to assess psychological distress. This may be explained by differences in the selection criteria, as well the presence of confounding factors such as the influence of the environment on the mental health of our participants, modulating both the individual’s subjective perception and the expression of symptoms of psychological discomfort. That is, it is possible that external factors including the participants’ geographical location as well as their sociocultural context can significantly affect the prevalence of psychological distress in this population.

Of our participants, 37.4% presented symptoms of two or more psychological disorders. This association has been previously described both in the general population [39], as well as in college students [11]. In fact, Long et al. [40] suggest that there is a bidirectional, systematic pattern between the development of depressive and anxious syndromes in young adults. In addition, previous studies [41,42,43] have identified similarities in the neurobiology and genetic structure of depression and anxiety. Another possible explanation for the association between depression, anxiety and stress is the fact that they share a significant number of risk factors and symptoms. Nevertheless, the reason for the association between these psychological syndromes is yet to be established.

In our sample, female students presented a higher prevalence of symptoms of stress and anxiety compared to male students. This is in agreement with previous studies, which also reported a higher prevalence of anxiety, stress and depression among women [39].

The relationship between lifestyle habits and mental health has been studied in detail. Thus, physical activity has often been associated with psychological wellbeing through a range of mechanisms including the secretion of endogen substances such as endorphins, the activity of the regulation of stress responses through the hypothalamic-pituitary-adrenal (HPA) axis, the improvement of sleep quality and the development of self-regulation and other coping mechanisms [44,45]. However, physical activity was not clearly associated with psychological distress in our sample. It seems reasonable to suggest that other factors, such as those linked to socialization, may have a bigger impact on mental health in adolescence and early adulthood, when personality is shaped and an adult role is gradually acquired.

Various possible explanations have been proposed to explain the relationship between tobacco and psychological distress in our population. First, it is likely that tobacco use and mental health problems have a common root; people who experience mental health problems may smoke to regulate feelings of low mood, stress and anxiety; smoking could also cause or exacerbate existing mental health problems [46]. With regard to the association between alcohol and psychological distress, several studies [47,48] have reported strong behavioral and neurologic interactions. In addition, drinking alcohol carries a heavy social component which, in part, may explain its intense consumption in youth and adolescence.

PIU has been studied in the past few years, both in the general population and, especially, in the young and adolescent. The negative consequences of PIU include, among others, a possible increase in stress and anxiety, as well as a lack of social communication and interaction [49,50]. This may seem paradoxical. However, even when used for social interaction, digital screen time can reduce the time spent developing skills to read and interpret cues of human emotion.

Recent studies [51,52] have reported a positive association between dietary quality and mental health problems. However, two previous systematic reviews [53,54] found limited and contradictory evidence to support the association between dietary patterns and mental health problems in adults. 

As has been reported in previous studies involving similar samples, we found an association between certain habits such as tobacco and alcohol consumption [32], presenting PIU [11] and eating unhealthily [43,55] with higher levels of psychological distress. It is important to highlight that, although we were not able to establish the direction of these associations, it seems reasonable to hypothesize that a bidirectional relationship exists between these variables. Thus, we suggest that future mental health promoting strategies in this population include an evaluation of lifestyle.

It is also worth highlighting that being enrolled in a healthcare program did not increase the risk of experiencing symptoms of depression, anxiety and stress in our sample. This is contrary to previous studies [56,57], which identified that being enrolled in a health-related program of study was a risk factor for psychological distress. They argue that health-related programs pose unique challenges including excessive academic pressure, peer competition, working under minimal supervision in unfamiliar clinical settings and witnessing pain, suffering and death [30].

It is possible that college students perceive vital events associated with college life as threatening to them, thus negatively affecting their mental health. For example, being under 21 was significantly associated with symptoms of depression, anxiety and stress [58]. This is an interesting finding, which may suggest that younger students may be more likely to experience uncertainty related to their studies than mature students. This observation is supported by previous studies [1,2], which reported higher levels of anxiety in the initial years of study.

This, to our knowledge, is the first attempt to establish an association between the symptoms of anxiety, depression and stress, and a large range of socioeconomic and behavioral variables, in a sample of Spanish college students. Furthermore, we believe that our results are highly representative of, and generalizable to, the population of college students in Spain, owing not only to our sample size but also to the standardized procedures of data collection and plausibility of the associations established. This has allowed us to draw a reliable picture of the psychological health and habits of our population of college students, which may serve as a starting point for the development and implementation of preventative and diagnostic interventions, and treatment services, to promote mental health and well-being.

Some authors [59,60] have argued that educational settings are ideal to implement measures and interventions leading to the promotion of a healthy lifestyle and prevention of mental health problems. Higher education institutions must attempt to train not only excellent professionals but also healthy individuals. Some Spanish universities, known as Health Promoting Universities, have accepted this challenge and are systematically implementing measures to promote health and wellbeing among the university population [61]. Future studies should compare the prevalence and level of psychological distress between the staff and the students enrolled in these universities, and those working and studying in non-health promoting ones.

This study has some limitations that need to be acknowledged. Firstly, the DASS-21 questionnaire is a suitable tool to screen for anxious, depressive and stress disorders, and may be useful to identify patients who are at risk of being affected by these conditions. However, additional tools should be used to establish a formal diagnosis. Secondly, the methodology employed in this investigation does allow for the establishment of associations between variables, but not causality. Finally, we would like to highlight that data collection took place over a period of eight months and, consequently, it is likely that academic life conditions were different for some of the students. For example, it is well known that factors such as the proximity of deadlines and exams may become a significant source of stress and anxiety for college students [62]. Furthermore, the generalizability of our results to the general population of Spanish college students may be limited. Specifically, this study was carried out in a private college in Spain. Such private higher education institutions are less numerous than public ones in Spain and tuition fees tend to be significantly higher. Having said this, the sociodemographic characteristics of our sample are similar to those of similar studies conducted in Spain involving college students enrolled in public universities [63,64]

Future investigations in this area should attempt to address these limitations. In any case, we argue that our results bring to light the need to implement strategies to protect and, if applicable, improve the mental health and wellbeing of college students.

## 5. Conclusions

We found a considerable prevalence of symptoms of depression, anxiety and stress in our population which, in some cases, do not occur in isolation, but coexist. In addition, we identified a number of factors associated with these symptoms. Factors including age, gender, self-esteem, sleep quality and living arrangements of college students, as well as specific behaviors relating to alcohol, tobacco and Internet use seem to be strongly associated with psychological distress in the college student population. We argue that our results can be helpful to design strategies for the early identification of mental health disorders, as well as psychological and other interventions leading to mental health promotion and wellbeing in the population of college students.

## Figures and Tables

**Table 1:** Participants’ characteristics (*n* = 1074).

Variables		*n* (%)
Age	Mean ± SD	21.73 ± 5.12
Age groups	<21 years	650 (60.5%)
	≥21 years	424 (39.5%)
Gender	Female	763 (71.0%)
	Male	311 (29.0%)
Type of bachelor’s degree	Healthcare	615 (57.3%)
	Other	459 (42.7%)
Place of residence	Lives alone or with friends	362 (33.7%)
	Lives with his/her family	712 (66.3%)
Stable partner	Yes	503 (46.8%)
	No	571 (53.2%)
Perceived financial status	Low	124 (11.5%)
	Medium	802 (74.7%)
	High	148 (13.8%)
BMI	Mean ± SD	21.4 ± 3.48
BMI categories	Low	163 (15.2%)
	Normal	750 (69.8%)
	High/obese	161 (15.0%)
Tobacco	Yes	267 (24.9%)
	No	807 (75.1%)
Alcohol consumption categories	Never or occasionally	754 (70.2%)
	Once a week	268 (25.0%)
	Twice a week	22 (2.0%)
	≥3 times/week	30 (2.8%)
Physical activity (MET * minutes/week)	Mean ± SD	2372.27 ± 2460.66
	Low physical activity	301 (28.0%)
	Moderate physical activity	457 (42.6%)
	High physical activity	316 (29.4%)
Internet Addiction Test score	Mean ± SD	41.75 ± 9.23
Use of Internet categories	No PIU	827 (77.0%)
	PIU	247 (23.0%)
Healthy Eating Index score	Mean ± SD	68.53 ± 12.16
Diet categories	Good/healthy	187 (17.4%)
	Needs improvement or unhealthy	887 (82.6%)
Insomnia Severity Index score	Mean ± SD	7.91 ± 4.87
Insomnia categories	No insomnia	613 (57.1%)
	Mild	337 (31.4%)
	Moderate	116 (10.8%)
	Severe	8 (0.7%)
Rosenberg Self-Esteem Scale score	Mean ± SD	32.09 ± 5.38
Self-esteem categories	Low	102 (9.5%)
	Medium	172 (16.0%)
	High	800 (74.5%)

* Metabolic equivalents (MET) are defined as “the ratio of a person’s working metabolic rate relative to their resting metabolic rate”.

**Table 2:** Total scores from the DASS-21 and by gender.

DASS-21 ^a^	Categories	Total (*n* = 1074)	Men (*n* = 311)	Women (*n* = 763)	*P*
DASS-D ^b^	No depression	876 (81.6%)	251 (80.7%)	625 (81.9%)	0.000
	Mild	81 (7.5%)	31 (10.0%)	50 (6.6%)	
	Moderate	48 (4.5%)	0 (0%)	48 (6.3%)	
	Severe	39 (3.6%)	14 (4.5%)	25 (3.3%)	
	Extremely severe	30 (2.8%)	15 (4.8%)	15 (2.0%)	
	Score. Mean ± SD	5.44 ± 7.09	5.44 ± 8.28	5.44 ± 6.55	NS *
DASS-A ^c^	No anxiety	820 (76.4%)	266 (85.5%)	554 (72.6%)	0.000
	Mild	87 (8.1%)	16 (5.1%)	71 (9.3%)	
	Moderate	96 (8.9%)	29 (9.3%)	67 (8.8%)	
	Severe	9 (0.8%)	0 (0%)	9 (1.2%)	
	Extremely severe	62 (5.8%)	0 (0%)	62 (8.1%)	
	Score. Mean ± SD	4.85 ± 5.74	3.35 ± 3.33	5.46 ± 6.41	0.000
DASS-S ^d^	No stress	704 (65.5%)	253 (81.4%)	451 (59.1%)	0.000
	Mild	125 (11.6%)	28 (9.0%)	97 (12.7%)	
	Moderate	181 (16.9%)	30 (9.6%)	151 (19.8%)	
	Severe	47 (6.2%)	0 (0%)	47 (6.2%)	
	Extremely severe	17 (2.2%)	0 (0%)	17 (2.2%)	
	Score. Mean ± SD	12.45 ± 8.07	9.80 ± 6.13	13.53 ± 8.51	0.000

* NS: Non-significant. (^a^) DASS-21: 21 item Depression, Anxiety Stress Scale. (^b^) DASS-D: 7-item DASS-21 Depression subscale. (^c^) DASS-A: 7-item DASS-21 Anxiety subscale. (^d^) DASS-S: 7-item DASS-21 Stress subscale.

**Table 3:** Factors associated with symptoms of depression, anxiety and stress in Spanish college students. OR ^a^ (IC95%).

Factors	Symptoms of Stress (DASS-S >14)		Symptoms of Depression (DASS-D >9)		Symptoms of Anxiety (DASS-A >7)	
	Bivariate Model	Multivariate Model	Bivariate Model	Multivariate Model	Bivariate Model	Multivariate Model
Age						
<21 years	Reference (OR = 1)		Reference		Reference	
≥21 years	0.53 (0.41; 0.70) *	0.36 (0.24; 0.53) *	0.23 (0.15; 0.35) *	0.42 (0.26; 0.68) *	0.50 (0.36; 0.68) *	0.55 (0.38; 0.80) *
Gender						
Male	Reference		Reference		Reference	
Female	3.01 (2.19; 4.15) *	1.86 (1.21; 2.84) *	0.92 (0.66; 1.29)		2,23 (1.56; 3.17) *	1.11 (0.73; 1.71)
Degree program						
Healthcare bachelor’s degree	Reference		Reference		Reference	
Other	0.84 (0.65; 1.08)	0.78 (0.56; 1.08)	0.93 (0.68; 1.27)		0.87 (0.65; 1.15)	
Living arrangement						
Lives alone or with friends	Reference		Reference		Reference	
Lives with his/her family	1.24 (0.95; 1.63)	1.41 (1.00; 1.99) ^†^	1.86 (1.30; 2.66) *	0.87 (0.54; 1.41)	1.60 (1.17; 2.20) *	0.92 (0.64; 1.32)
Relationship stability						
Stable partner	Reference		Reference		Reference	
No stable partner	0.40 (0.31; 0.52) *	0.39 (0.28; 0.56) *	1.41 (1.03; 1.93) ^†^	2.28 (1.45; 3.59) *	0.71 (0.54; 0.95) ^†^	0.76 (0.53; 1.08)
Perceived financial status						
Low perceived financial status	Reference		Reference		Reference	
Medium/high perceived financial status	1.32 (0.88; 1.99)	1.67 (0.94; 2.95)	1.88 (1.05; 3.36) ^†^	1.27 (0.55; 2.92)	1.49 (0.92; 2.43)	1.18 (0.64; 2.15)
BMI						
Low BMI	1.13 (0.79; 1.61)		1.10 (0.71; 1.69)		1.00 (0.67; 1.49)	
Normal BMI	Reference		Reference		Reference	
High BMI/obese	1.04 (0.72; 1.48)		1.03 (0.66; 1.60)		0.91 (0.61; 1.38)	
Alcohol						
No/occasional alcohol consumption	Reference					
			Reference		Reference	
Regular alcohol consumption	1.51 (1.13; 2.00) *	3.19 (2.12; 4.80) *	1.01 (0.71; 1.40)		1.51 (1.08; 2.08) ^†^	2.59 (1.74; 3.87) *
PUI ^b^						
No PUI	Reference		Reference		Reference	
PUI	3.55 (2.66; 4.77) *	3.23 (2.22; 4.70) *	4.51 (3.25; 6.28) *	3.32 (2.14; 5.13) *	4.42 (3.24; 6.02) *	2.97 (2.09; 4.23) *
Tobacco						
Non-smoker	Reference		Reference		Reference	
Smoker	1.74 (1.31; 2.31) *	1.78 (1.19; 2.67) *	1.94 (1.39; 2.70) *	2.50 (1.58; 3.96) *	1.85 (1.36; 2.51) *	2.15 (1.45; 3.19) *
Physical activity						
					Reference		
Low physical activity	Reference		Reference			
Moderate/high physical activity	0.81 (0.62; 1.05)	0.95 (0.67; 1.34)	0.53 (0.39; 0.73) *	1.02 (0.66; 1.58)	0.59 (0.44; 0.79) *	0.77 (0.55; 1.08)
Diet						
Good/healthy diet	Reference		Reference		Reference	
Needs improvement or unhealthy diet	1.70 (1.23; 2.35) *	4.44 (2.91; 6.79) *	1.22 (0.82; 1.81)		1.20 (0.81; 1.75)	
Insomnia						
No insomnia	Reference		Reference		Reference	
Insomnia	6.03 (4.57; 7.96) *	4.77 (3.41; 6.68) *	5.67 (3.98; 8.08) *	2.86 (1.82; 4.48) *	3.59 (2.67; 4.84) *	2.15 (1.52; 3.03) *
Self-esteem						
Normal/high self-esteem	Reference		Reference		Reference	
Low self-esteem	5.15 (3.30; 8.03) *	3.01 (1.72; 5.26) *	38.0 (21.7; 66.3) *	26.3 (14.22; 48.8) *	3.30 (2.17; 5.02) *	2.04 (1.24; 3.35) *

* *p* < 0.01; ^†^ *p* < 0.05. Non-significant variables according to the following criterion: probability value for the entry of *p* = 0.05 and removal of *p* = 0.1, were removed from the model after each step. The values from the variables that remained after the last step of the model are shown in the multivariable model columns. (^a^) OR: Odds ratio. (^b^) PUI: Problematic use of the internet.
